# Association between visceral adipose surrogates and infertility among reproductive-aged females: a cross-sectional study

**DOI:** 10.3389/fendo.2024.1488309

**Published:** 2024-12-12

**Authors:** Dongli Guo, Ruiya Wang

**Affiliations:** Department of Physiological Obstetrics, Women and Children’s Hospital, Zhumadian Central Hospital, Zhumadian, Henan, China

**Keywords:** visceral adipose tissue, female infertility, NHANES, biomarkers, obesity

## Abstract

**Background:**

Visceral adipose tissue (VAT) exerts a substantial influence on female infertility. Nevertheless, the relationship between VAT surrogates and female infertility remains ambiguous.

**Methods:**

This study employed a cross-sectional design and analyzed data from the National Health and Nutrition Examination Survey (NHANES, 2012–2016). Weighted logistic regression models were utilized to examine the association between VAT surrogates and infertility. Furthermore, Receiver Operating Characteristic (ROC) curve analysis was conducted to assess the diagnostic efficacy of these surrogates for infertility.

**Results:**

Individuals experiencing infertility exhibited markedly elevated levels of the Chinese Visceral Adiposity Index (CVAI) (108.30 vs. 69.86, *P*<0.001) and Visceral Adiposity Index (VAI) (1.68 vs. 1.35, *P*<0.001). When considered as a continuous variable, CVAI (odds ratio [OR]: 1.06, 95% confidence interval [CI]: 1.03-1.09, *P*<0.001), rather than VAI (OR:1.02, 95%CI: 0.98-1.06, *P*=0.259), demonstrated a significant association with the risk of female infertility. Consistent findings were also evident after dividing participants into 4 subgroups based on CVAI quartiles. Additionally, ROC curves indicated that CVAI exhibited the most robust diagnostic value for female infertility compared to other indices. Subgroup analyses revealed a robust association between CVAI and infertility across different populations.

**Conclusion:**

Females with elevated CVAI levels faced a significantly heightened risk of infertility in the United States. CVAI holds promise as a valuable tool for stratifying the risk of infertility.

## Introduction

Infertility, characterized by the inability to conceive after 12 months or more of unprotected sexual intercourse, is a reproductive disorder affecting both males and females (1). Current estimates indicate that approximately 186 million individuals worldwide suffer from infertility, with around one in seven couples in developed countries and one in four couples in developing countries affected by its impact (2, 3). Notably, female infertility accounts for approximately 40% of all reported cases. The prevalence of infertility poses a significant challenge to human development, leading the US Centers for Disease Control and Prevention (CDC) to recommend prioritizing its diagnosis and treatment (4).

Female infertility, a complex and multifaceted reproductive disorder, can arise from various factors, including dysregulated ovarian function, tubal infection, cervical factors, endometriosis, decreased ovarian reserve, uterine pathologies, and unexplained infertility (5, 6). The etiology of these infertility conditions can be influenced by genetic predisposition, infectious agents, environmental factors, and lifestyle factors like smoking and obesity (7). Notably, among these factors, obesity has been extensively investigated and shown to exert significant negative effects on reproductive health (8).

With rapid economic development and improved quality of life, the global rise in obesity, defined by excessive adipose tissue deposition, has become a growing public health concern (9). In 2016, the obesity rate among adult women in the US was 41.1% (10). Currently, body mass index (BMI) and waist circumference (WC) serve as common surrogates for obesity assessment; however, it is critical to acknowledge their limitations. BMI fails to differentiate between muscle and fat mass or distinguish between peripheral and abdominal fat, while WC measurements are influenced by an individual’s height and weight. The Visceral adipose index (VAI) and the Chinese VAI (CVAI), initially developed from Caucasians and Chinese populations, respectively, are cost-effective surrogates for evaluating visceral adipose tissue (VAT), which has been found more strongly associated with various diseases, such as hypertension, diabetes, and metabolic syndrome compared to subcutaneous adipose tissue (11, 12). CVAI, which is calculated using parameters such as age, BMI, WC, total triglycerides (TG), and high-density lipoprotein cholesterol (HDL-C), has been found to be more closely correlated with metabolic disorders than VAI. Previous research has linked higher BMI and WC levels to increased infertility risk in reproductive-age females (13, 14), yet the potential association between VAI/CVAI and female infertility remains unclear. Therefore, our study aims to explore the potential relationship between VAT indices and infertility and compare their diagnostic efficacy with BMI and WC in reproductive-aged females.

## Methods

### Study design and population

This study was cross-sectional and utilized data from the National Health and Nutrition Examination Survey (NHANES). This study included female participants aged 18 to 45 years from the 2012-2013, 2013-2014, and 2015-2016 survey cycles. Participants were excluded if they met any of the following criteria: (1) missing data on self-reported infertility; (2) without data on CVAI and VAI; (3) history of bilateral oophorectomy; (4) history of hysterectomy; (5) pregnancy at the time of examination.

The NHANES, conducted by the National Center for Health Statistics (NCHS) and the Centers for Disease Control and Prevention (CDC), employs a stratified and multistage probability sampling method to assess the health and nutritional status of the non-institutionalized civilian population of the United States every two years. Data is collected through standard questionnaires and physical examinations.

### Ethics approval and informed consent

Before conducting the survey, NHANES protocols were reviewed and approved by the Research Ethics Review Board of the NCHS. All participants provided written informed consent before participating in the study.

### Infertility definition

The primary outcome of this study is female infertility, which was identified using the following standard questionnaire item: “Have you tried for a year to become pregnant?” Participants responding “Yes” were categorized as “infertile”.

### Covariate variables

Additional covariate variables, including demographic data (age, race/ethnicity, education levels, marital status), blood pressure (BP) levels, poverty-income ratio, lifestyle characteristics (smoking status), and laboratory test results [serum fasting glucose, creatinine, total cholesterol (TC), TG, HDL-C, glycohemoglobin (HbA1c)], as well as information on hypertension and diabetes status, were collected based on clinical significance and previous literature. Hypertension was defined by meeting at least one of the following conditions: (1) self-reported, (2) taking anti-hypertensive agents, (3) systolic BP (SBP) ≥ 140mmHg, (4) diastolic BP (DBP) ≥ 90mmHg. Diabetes was defined by meeting at least one of the following conditions: (1) fasting glucose levels ≥ 7.0 mmol/L, (2) self-reported doctor diagnosis, (3) use of oral glucose-lowering medications or insulin, (4) HbA1c levels ≥ 6.5%.

### Calculation of variables


$$\text{BMI}=\text{weight}(\text{kg})/\text{heigh}{\text{t}}^{2}(\text{m})$$

$$\begin{array}{c} \text{VAT}=[\text{WC}(\text{cm})/(36.58+(1.89\times \text{BMI}(\text{kg}/\text{m}2))]\\ \times (\text{TG}(\text{mmol}/\text{L})/0.81)\times (1.52/\text{HDL}(\text{mmol}/\text{L})) \end{array}$$

$$\begin{array}{c} \text{CVAT}=-187.32+1.71\times \text{age}(\text{y})+4.32\times \text{BMI}(\text{kg}/\text{m}2)+1.12\\ \times \text{WC}(\text{cm})+39.76\times \text{LgTG}(\text{mmol}/\text{L})-11.66\\ \times \text{HDL}-\text{C}(\text{mmol}/\text{L}) \end{array}$$

$$\begin{array}{c} \text{TyG}-\text{BMI}:\text{Ln}[\text{fastingtriglycerides}(\text{mg}/\text{dl})\\ \times \text{fastingglucose}(\text{mg}/\text{dl})/2]\times \text{BMI} \end{array}$$

### Statistical analysis

In accordance with NCHS guidelines, this study followed established analytical protocols and applied sample weights during data analysis process. Continuous variables were presented as mean ± standard deviation (SD) or median (interquartile range, IQR) and tested using weighted t-test or Wilcoxon rank-sum test, as appropriate. Categorical variables were presented as percentages (%) and compared using weighted Chi-square test.

Three weighted logistic models were employed to evaluate the association between infertility and visceral adipose indices. Model I was a crude model without any adjustment, Model II was adjusted for age, race, education levels, poverty-income ratio, and smoking status, and Model III was further adjusted for marital status, hypertension, diabetes, TC, estimated glomerular filtration rate (e-GFR), SBP, DBP, and fasting glucose. Participants were categorized into four subgroups according to CVAI quartiles based on the logistic analysis results: Q1 (CVAI ≤ 30.20), Q2 (30.20 < CVAI < 73.25), Q3 (73.25 < CVAI < 125.54), and Q4 (CVAI ≥ 125.54). The risk for infertility among different groups was further evaluated. Additionally, a restricted cubic spline (RCS) curve was used based on Model III to explore any non-linear relationship between CVAI and infertility, with 4 knots of the quartiles. Receiver operating characteristic (ROC) curves were drawn to assess the diagnostic value of those indices for infertility, and differences between the AUCs were tested using the Z-test.

Interaction and subgroup analyses stratified by age, marital status, smoking status, education levels, hypertension, and diabetes were conducted to assess the robustness of the association.

All analyses in this study were performed using the R package (version 4.0.3), and a P-value < 0.05 was considered statistically significant.

## Results

### Baseline characteristics comparison between participants with and without infertility

This study included 3073 participants with an average age of 31.08 ± 8.37 years. The weighted prevalence of infertility was 11.04%. **Table 1** presents the comparison of baseline characteristics between individuals with and without infertility. Individuals in the infertility group tended to be older, married or living with a partner, have higher poverty-income ratios, be smokers, have hypertension and diabetes, and have higher levels of TC, fasting glucose, VAI, and CVAI. They also had lower levels of eGFR.

**Table 1:** Baseline characteristics of the participants.

Variables	Overall (N=3073)	Non-infertility(N=2758)	Infertility (N = 315)	*P*-Value
Age (years)	31.08±8.37	30.65±8.40	34.76±7.12	<0.001
Race/ethnicity (%)				0.242
Mexican American	12.45	12.55	11.70	
Other Hispanic	8.06	8.32	6.03	
Non-Hispanic White	56.11	55.62	60.08	
Non-Hispanic Black	12.71	12.69	12.80	
Other Race	10.66	10.82	9.38	
Marital status (%)				<0.001
Married or Living with partner	59.25	57.03	76.04	
Other	40.75	42.97	23.96	
Education level (%)				0.227
Less than high school	12.17	12.29	11.24	
High school or above	87.83	87.71	88.76	
PIR	2.29±1.60	2.26±1.59	2.51±1.65	0.012
Smoking status (%)				0.001
Current	18.59	18.12	22.41	
Former	11.53	11.17	14.45	
Never	69.88	70.71	63.14	
Hypertension (%)	13.65	12.76	20.85	<0.001
Diabetes	5.80	5.35	9.47	<0.001
TC (mmol/L)	4.64±0.91	4.63±0.91	4.76±0.92	0.018
Fasting glucose (mmol/L)	5.15±1.40	5.12±1.28	5.40±2.16	<0.001
e-GFR (ml/min/1.73m2)	105.93±24.86	106.26±25.05	103.07±22.98	0.031
VAI	1.38 (0.86, 2.40)	1.35 (0.84, 2.36)	1.68 (1.09, 3.03)	<0.001
CVAI	73.24 (30.20, 125.54)	69.86 (28.02, 121.24)	108.30 (59.58, 153.42)	<0.001

PIR, ratio of family income to poverty; TC, total cholesterol; e-GFR, estimated glomerular filtration rate; VAI, visceral adiposity index; CVAI, Chinese visceral adiposity index.

### Association between different VAT indices and female infertility

**Figure 1:**
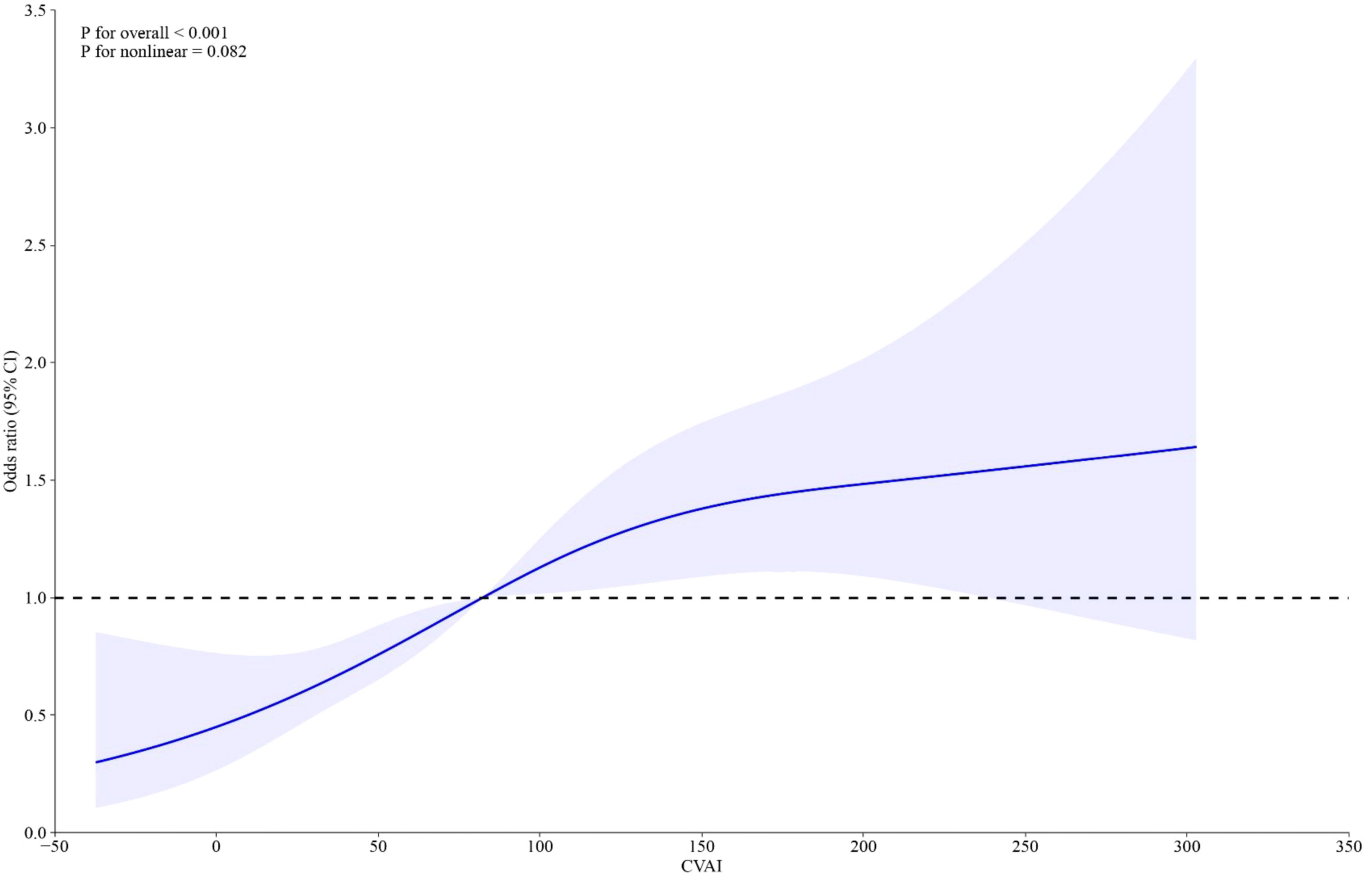
RCS fitting for the dose-response association between CVAI with female infertility.

**Figure 2:**
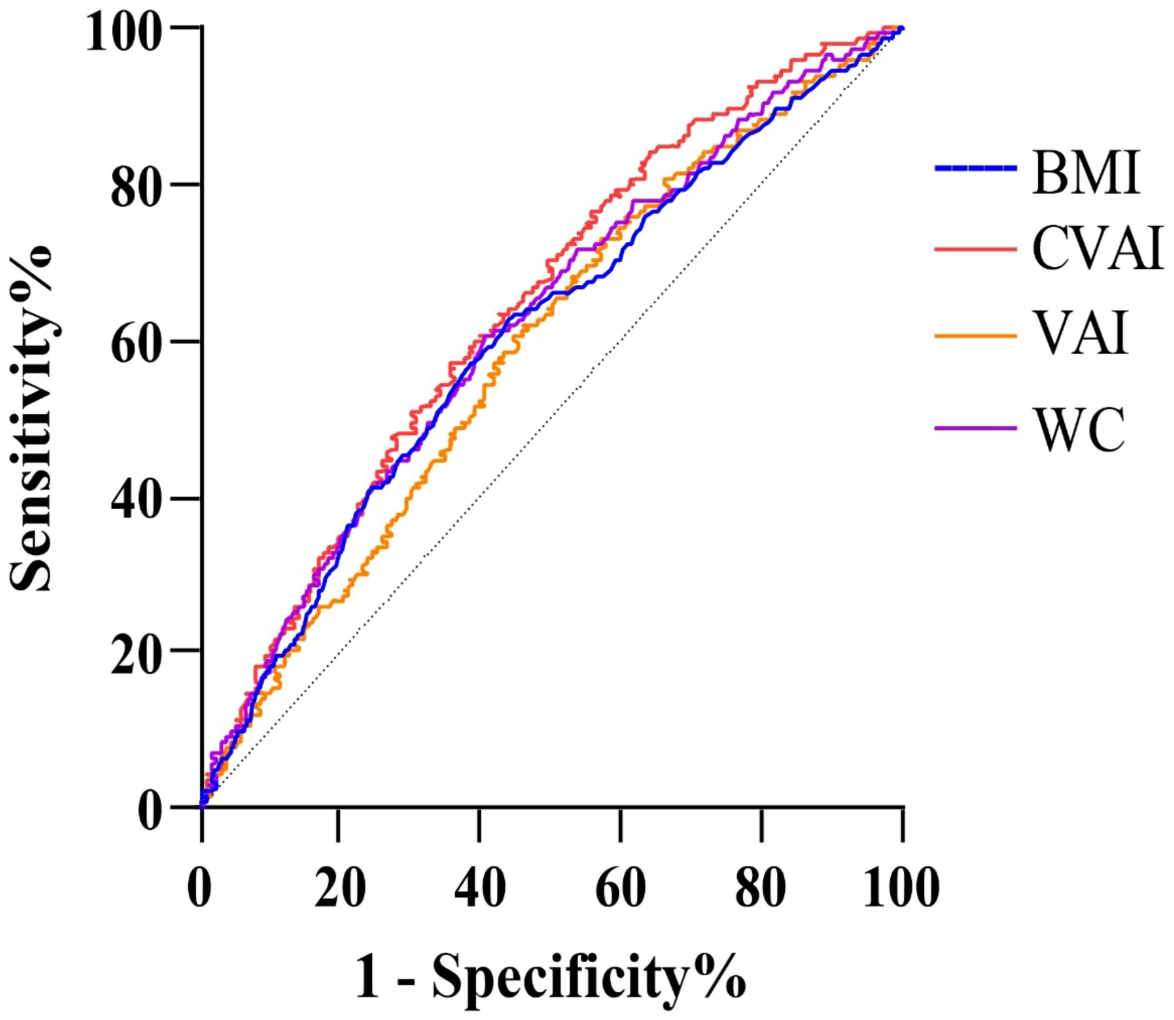
ROC for different indices for predicting female infertility.

Weighted logistic models assessed the association between VAT indices and infertility, as shown in **Table 2**. After adjusting for potential confounding risk factors, CVAI was significantly associated with an increased risk of infertility, while VAI did not show a significant association. Further analysis categorized participants into quartiles of CVAI. Participants in the highest CVAI quartile (Q4) had a 3.73-fold increased infertility risk (odds ratio (OR) 3.73, 95% confidence interval (CI) 2.07-6.74, *P*<0.001) compared to those in the lowest quartile (Q1). A restricted cubic spline curve demonstrated a linear dose-response relationship between CVAI and the risk of infertility (**Figure 1**). ROC curve analysis revealed that BMI, WC, CVAI, and VAI demonstrated significant diagnostic value for infertility. The area under the curve (AUC) analysis indicated that CVAI had the strongest diagnostic value for the infertility among the indices (CVAI: 0.65 vs. BMI 0.60 vs. WC 0.61 vs. VAI 0.59, *P*<0.001) (**Figure 2**).

**Table 2:** The association between different indices and infertility.

	Model I	Model II	Model III
BMI	1.04 (1.03-1.06) <0.001	1.04 (1.02-1.05) <0.001	1.04 (1.02-1.06) <0.001
WC	1.02 (1.01-1.03) <0.001	1.02 (1.01-1.03) <0.001	1.02 (1.01-1.03) <0.001
CVAI	1.07 (1.05-1.09) <0.001	1.06 (1.03-1.08) <0.001	1.06 (1.03-1.09) <0.001
VAI	1.04 (0.94-1.15) 0.457	1.04 (0.96-1.11) 0.347	1.02 (0.98-1.06) 0.259
CVAI quartiles			
Q1 (≤30.20)	1.00	1.00	1.00
Q2 (30.20-73.25)	2.77 (1.65-4.66) <0.001	2.11 (1.19-3.72) 0.010	1.99 (1.10-3.59) 0.023
Q3 (73.25-125.54)	3.25 (1.98-5.36) <0.001	2.56 (1.44-4.55) 0.001	2.38 (1.32-4.30) 0.004
Q4 (≥125.54)	5.29 (3.28-8.53) <0.001	3.74 (2.13-6.59) <0.001	3.73 (2.07-6.74) <0.001
*P* for trend	<0.001	<0.001	<0.001

Data were present as odds ratio (OR), 95% confidence interval (CI) and P value; Model I was adjusted for none; Model II was adjusted for age, race, education levels, smoking status, ratio for the income to poverty; Model III was further adjusted for marital status, hypertension, diabetes, total cholesterol, estimated glomerular filtration rate, systolic blood pressure, diastolic blood pressure and fasting glucose.

### Subgroup analysis

**Figure 3:**
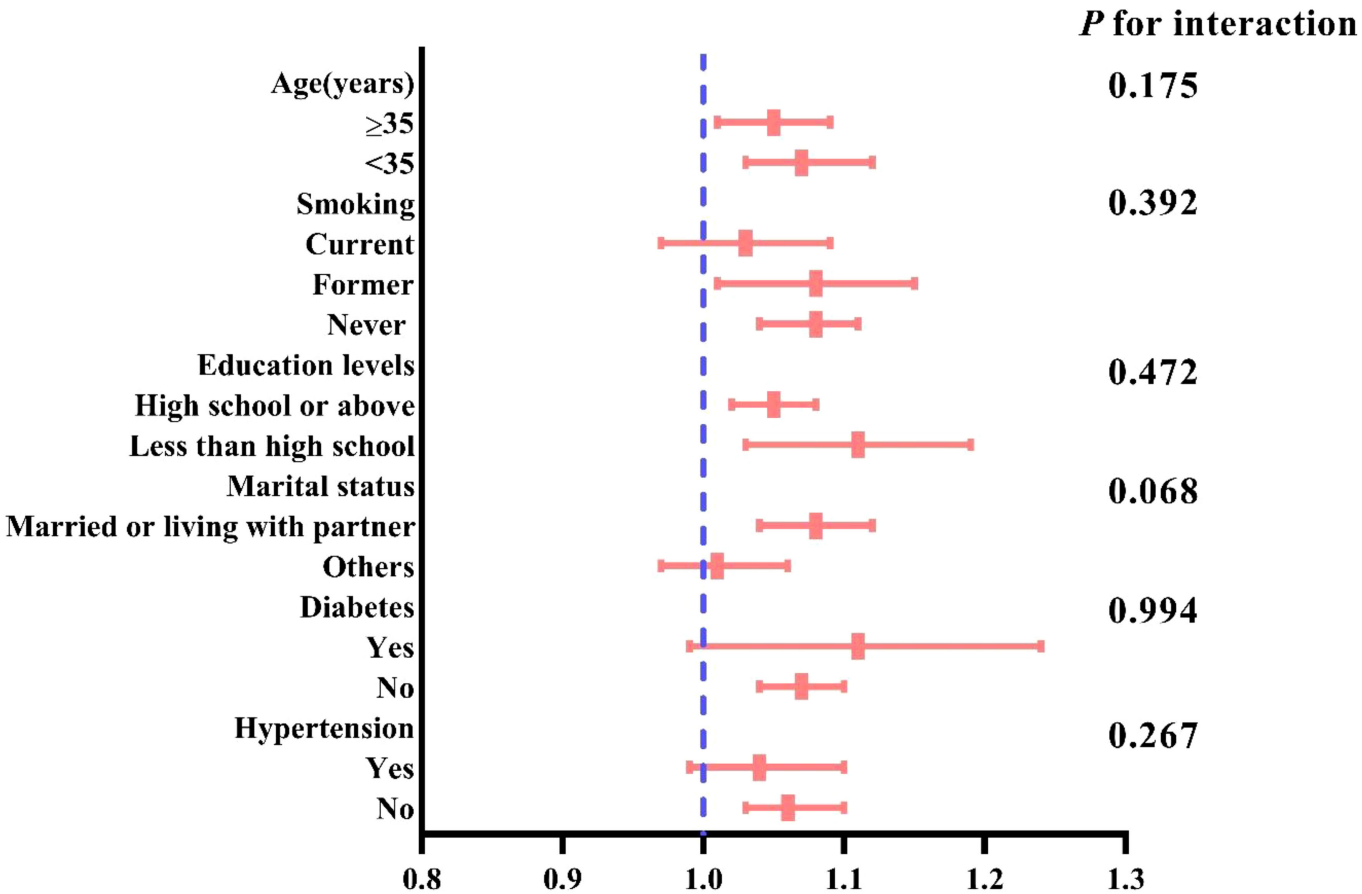
Subgroup analysis for the association between CVAI and female infertility.

Subgroup analysis, stratified by age smoking status, education level, marital status, hypertension, and diabetes, assessed the robustness of the association between CVAI and female infertility. The results indicated a consistent, significant association between CVAI and infertility across all subgroups (*P* for interaction>0.05) (**Figure 3**).

## Discussion

To our knowledge, this study is the first to investigate the potential association between surrogate measures of VAT and female infertility in reproductive-aged women. The main findings of this study are as follows: (1) CVAI, rather than VAI, shows a significant association with infertility and has the strongest diagnostic value among the indices tested; (2) A linear, positive dose-response relationship exists between CVAI and the risk of female infertility.

In this study, we found that the prevalence of infertility among women of reproductive age in the United States was 11.04% in a large-scale, nationally representative cohort, which is consistent with previous reports (15).

Polycystic ovary syndrome (PCOS), a hormonal disorder characterized by irregular menstrual cycles, polycystic ovaries, and hyperandrogenism, is a significant pathological and physiological factor associated with female infertility (16, 17). Studies indicate that approximately 40% of women diagnosed with PCOS experience infertility. In fact, PCOS is considered the leading cause of anovulatory infertility, where the ovaries do not release eggs regularly. Reports indicate that a high percentage, ranging from 90% to 95%, of women with anovulatory infertility seeking treatment at infertility clinics are diagnosed with PCOS (18). The prevalence of PCOS varies depending on the specific diagnostic criteria used. When employing the diagnostic criteria outlined by the European Society for Human Reproduction and Embryology/American Society for Reproductive Medicine, the prevalence of PCOS has been observed to reach levels as high as 15%-20% (19).

Accumulating evidence has confirmed a robust association between obesity and PCOS (20). However, VAT, rather than subcutaneous fat, is significantly linked to the hormonal imbalances seen in PCOS, which notably impact female fertility (19). Computed tomography (CT) and magnetic resonance imaging (MRI) are recognized as gold standard methods for accurately measuring VAT. However, due to the high costs and potential radiation risks, these methods are not routinely used as clinical screening tests, particularly for women planning pregnancy. VAI and CVAI are indices derived from Western and Chinese populations, respectively, using easily obtainable indicators in routine clinical practice to evaluate VAT levels (11–21). Previous studies have confirmed a strong association between these indices and various diseases and metabolic disorders, such as insulin resistance, hypertension, and diabetes (22, 23). In this study, we found a significant positive linear dose-response relationship between CVAI and infertility, while no such significant association was observed for VAI. Compared to VAI, CVAI is a more comprehensive indicator that considers age, which is significantly associated with VAT deposition. Furthermore, we also evaluated the potential association between CVAI and two well-known surrogate markers of insulin resistance, TyG index and TyG-BMI index, and found a positive association with both indices (**Supplementary Figure S1**). Thus, in this study, we focused on CVAI because it directly reflects the role of visceral fat in metabolic and reproductive health. Elevated CVAI, which is a surrogate for visceral adiposity, is associated with a higher risk of metabolic conditions such as insulin resistance, hypertension, and dyslipidemia, all of which are implicated in infertility. By specifically examining CVAI, we can better isolate the impact of visceral fat on fertility outcomes, as opposed to general obesity or diabetes, which are broader and less specific markers of metabolic dysfunction.

Obesity, particularly central obesity, further exacerbates the metabolic and reproductive abnormalities associated with PCOS (24). Women with both PCOS and obesity face a heightened risk of developing insulin resistance, hyperinsulinemia, cardiovascular disease (CVD), and type 2 diabetes (25, 26). Among obese females, increased circulating insulin has been shown to stimulate the synthesis of androgens, which can be converted into estrogen as a response to excess adipose tissue. This leads to negative feedback on the hypothalamic-pituitary-ovarian (HPO) axis, resulting in infertility (27). Beyond their role in energy storage, adipose tissues are also recognized as important endocrine organs, secreting various bioactive cytokines, or adipokines, such as leptin, adiponectin (APN), resistin, visfatin, and omentin, as well as other cytokines like retinol binding protein-4 (RBP4), lipocalin-2 (LCN2), chemerin, interleukin-6 (IL6), interleukin-1β (IL1β), and tumor necrosis factor-α (TNFα). These cytokines regulate various physiological functions including reproduction, immune response, and glucose and lipid metabolism (28). Leptin, secreted by white adipose tissue (a component of VAT), has been found to significantly influence gonadotropin-releasing hormone (GnRH) pulse neurons and the production of luteinizing hormone (LH) and follicle-stimulating hormone (FSH) from the pituitary (29).

Lipid metabolism disorders are common in individuals with obesity and include increased levels of TG and low-density lipoprotein-cholesterol (LDL-C), as well as reduced HDL-C. Dyslipidemia not only affects the cardiovascular system but also the reproductive system. Excessive lipid accumulation in ovarian granulosa cells can cause lipo-toxicity, leading to cellular stress through interconnected pathways. The accumulation of lipids enhances the production of reactive oxygen species (ROS), resulting in oxidative stress (30). At the same time, this lipid overload disrupts the function of the endoplasmic reticulum (ER), triggering ER stress and activating the unfolded protein response, which may potentially lead to apoptosis if left unresolved (31).

In this study, CVAI quantifies visceral fat, a metabolically active tissue distinct from subcutaneous fat, which plays a crucial role in systemic inflammation and metabolic health. Visceral fat is a source of pro-inflammatory cytokines and adipokines, contributing to systemic inflammation and insulin resistance (32, 33), both contribute to type 2 diabetes. Insulin resistance associated with higher visceral adiposity can disrupt ovarian function by altering the balance of reproductive hormones. This disruption is particularly relevant in conditions like PCOS, where the hypothalamic-pituitary-gonadal axis is affected, often resulting in female infertility (34). Central obesity, resulting by excessive VAT deposition, exacerbates these hormonal imbalances. Excessive VAT elevates androgen-to-estrogen conversion rates, disrupting the hypothalamic-pituitary-gonadal axis and impacting ovulatory cycles (35). Furthermore, VAT’s effects on sex hormone-binding globulin (SHBG) and insulin can impair follicular development, which is crucial for female fertility (36, 37). Additionally, CVAI-related insulin resistance also significantly contributed to the development of hypertension through various mechanisms such as endothelial dysfunction, increased renal sodium reabsorption, sympathetic activity, and vascular hypertrophy (38). A recent population-based cohort study found that hypertension, no matter of controlled or uncontrolled, was significantly associated with decreased fecundability (39). An animal model study showed that renovascular hypertension induces an overall decrease in reproductive function in female rats (40). However, the exact mechanism and etiological pathway underlying the relationship between maternal hypertension and fecund ability have yet to be elucidated. Hypertension may impair uterine blood flow, reducing endometrial receptivity and affecting embryo implantation. Endothelial dysfunction can lead to compromised placental development, increasing the risk of early pregnancy complications and loss (41). These interconnected pathways underscore VAT’s importance, as reflected by CVAI in this study, as a pivotal factor linking metabolic disturbances to reproductive health challenges. Interventions aimed at reducing visceral fat, hence lowering CVAI, may offer a potential strategy to mitigate infertility risks associated with these metabolic and vascular conditions.

Some limitations in this study should be pointed out. The cross-sectional nature of the NHANES survey employed in our study precludes us from establishing a causal relationship between CVAI and female infertility. Additionally, the use of self-reported data to measure infertility may introduce recall bias, as participants may have trouble in accurately recalling the duration of their attempts to conceive. While we undertook measures to control for potential confounding variables, the possibility of residual confounding cannot be entirely ruled out. Furthermore, the findings of our study are limited to the United States population and may not be generalizable to other countries or populations with differing socio-demographic, cultural, and environmental characteristics.

## Conclusion

As surrogates for the VAT, CVAI, rather than VAI, was positively associated with the risk of female infertility with reproductive age in the United States.

## Data Availability

The original contributions presented in the study are included in the article/**Supplementary Material**. Further inquiries can be directed to the corresponding author.
