# Supplementary figures and images for: Association between visceral adipose surrogates and infertility among reproductive-aged females: a cross-sectional study
Source: Front Endocrinol (Lausanne). 2024 Dec 12;15:1488309. doi: 10.3389/fendo.2024.1488309 (PMC11669517; doi:10.3389/fendo.2024.1488309)

Figure S1: Association between CVAI and TyG index (A) and TyG-BMI index (B).


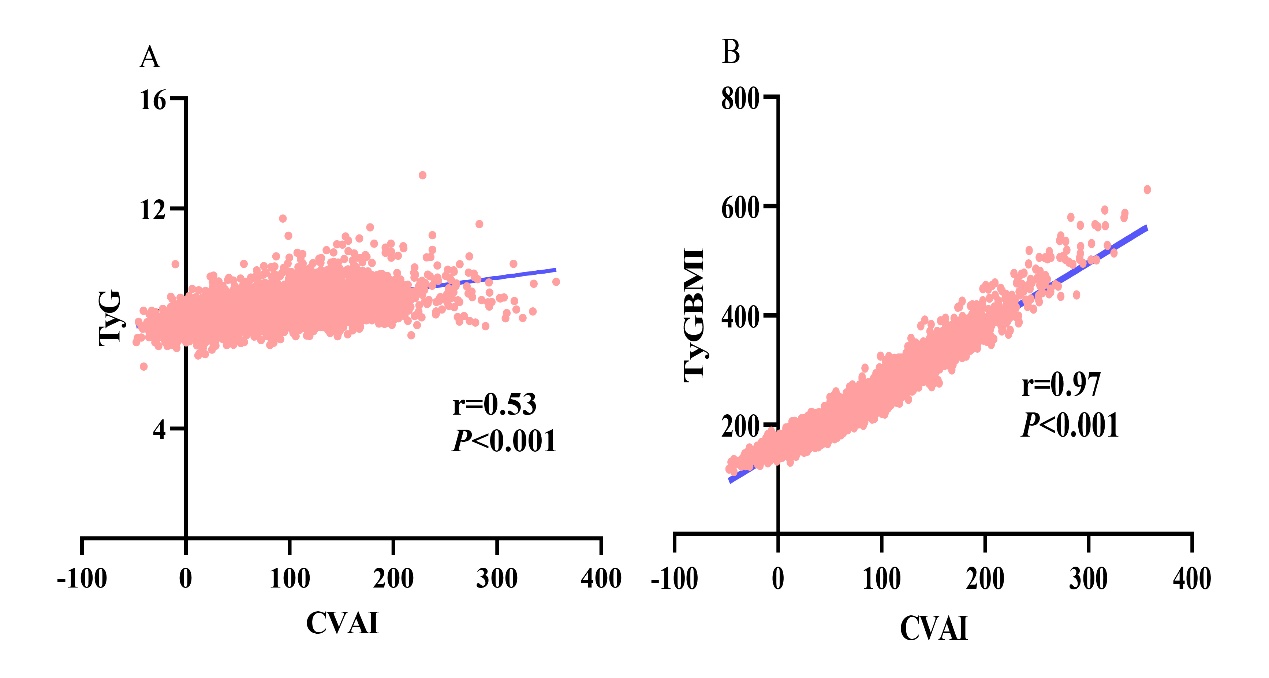

Supplement: Supplementary file 1 [file DataSheet1.docx]
